# Neonatal outcome of children born after ICSI with epididymal or testicular sperm: A 10-year study in China

**DOI:** 10.1038/s41598-020-62102-y

**Published:** 2020-03-20

**Authors:** Lei Jin, Zhou Li, Longjie Gu, Bo Huang

**Affiliations:** Reproductive Medicine Center, Tongji Hospital, Tongji Medicine College, Huazhong University of Science and Technology, Wuhan, People’s Republic of China

**Keywords:** Reproductive techniques, Embryology

## Abstract

Some studies show that children born after ICSI with non-ejaculated sperm are at increased risk of birth defects, other studies hold the opposite view. Does neonatal outcome including congenital malformations in children born after ICSI with percutaneous epididymal sperm aspiration (PESA) and testicular sperm aspiration (TESA) differ from neonatal outcome in children born after ICSI with ejaculated sperm? In this study, we examined the data from our IVF center from 2006 to 2016, to compare neonatal outcomes and rates of congenital malformations in children born after ICSI with different sperm origin. The results showed the clinical pregnancy rate and implantation rate of non-ejaculated sperm group were significantly higher (P < 0.001) than ejaculated sperm group. There were 775 clinical pregnancies from non-ejaculated sperm group and 2,486 clinical pregnancies from ejaculated sperm group. Most of the clinical pregnancy outcomes were comparable between non-ejaculated sperm group and ejaculated sperm group (p > 0.05): the miscarriage rate per transfer, ectopic pregnancy rate per clinical pregnancy, induced abortion rate per clinical pregnancy and fetal deaths per clinical pregnancy. However, the live delivery rate per transfer of non-ejaculated sperm group was significantly higher than that of ejaculated sperm group (45.4% vs 36.7%, P < 0.001). Moreover, the comparison between the epididymal sperm, testicular sperm and ejaculated sperm groups showed there were no difference in the incidence of congenital malformations of babies live birth. Among singleton gestation live births, there were more girls than boys in both non-ejaculated sperm and ejaculated sperm group. In conclusion, the present study clearly showed no statistical increased risk in neonatal outcomes of newborns were found in the ICSI treatment with epididymal or testicular sperm. It may provide information for consultation for ICSI treatment in PESA or TESA patients.

## Introduction

Since the first successful *in vitro* fertilization-embryo transfer (IVF-ET) in the United Kingdom in 1978^1^ and first introduction of intracytoplasmic sperm injection (ICSI) in 1992^2^, an increasing number of infertile couples have babies through modern reproductive therapy.

Azoospermia is found in about 5% of infertile couples^3^ and is present in 10% of male infertility^4–6^. Since the introduction of ICSI, it is possible for infertile couples in case of azoospermia to father their own progeny by using sperm retrieved by percutaneous epididymal sperm aspiration (PESA) or testicular sperm aspiration (TESA). In 1994, ICSI with spermatozoa obtained from the testicle by either TESA or from the epididymis by PESA was reported^7–9^. In mainland China, the first baby of ICSI was born in 1996. From then on, ICSI with non-ejaculated sperm has gradually been used in reproductive centers throughout the country.

There is, however, with the widespread use of these techniques, concerns that the quality of spermatozoa in terms of DNA damage or maturation when collected from non-ejaculated semen may differ from that collected from ejaculated. In other words, it is also questioned whether sperm of different origins will affect the neonatal outcome and safety of ICSI. Therefore, concerns about the health of the children born after the use of non-ejaculated sperm have been raised. In 2010, a systematic review^10^ about congenital anomalies reported that there were no statistical difference in malformation rates in children after ICSI using non-ejaculated sperm compared with ejaculated sperm. However, it was suggested that more data are necessary to focus on the neonatal outcome of children born after ICSI with epididymal or testicular sperm.In recent years, some studies have shown the clinical and neonatal outcome in children born after ICSI with testicular or epididymal sperm^11–14^. These studies found that ICSI with non-ejaculated sperm does not lead to more stillbirths or congenital malformations in comparison to ICSI with ejaculated sperm in Belgium, Denmark, Norway and The Netherlands. Data in mainland China with a very large population are still lacking.

In this study, we examined the data from our IVF center from 2006 to 2016, to compare neonatal outcomes and rates of congenital malformations in children born after ICSI with different sperm origin.

## Materials and Methods

### Patients

This was a noninterventional, retrospective, single-center cohort study of patientstreated with one or more cycles at the Reproductive Medicine Center of Tongji Hospital between January 2006 and December 2016. A total of 10,520 patients undergoing PESA, TESA and conventional ICSI were enrolled. For PESA and TESA groups, patients with an azoospermia were seen by urologists to determined whether there was an obstructive azoospermia (OA) or non-obstructive azoospermia (NOA), according to anamnesis for obstruction, volume of testis, hormonal level. On the day of oocyte retrival,

PESA was performed in OA patients, and TESA was performed when sperm was not recovered by PESA. In the NOA patients, TESA was performed directly. For ICSI group, patients with severe oligozoospermia (≤5 million/mL), conventional ICSI were conducted with ejaculated sperm. All patients of this study gave written informed consent. This included the information of total fertilization failure or poor fertilization, and follow-up regarding pregnancy, birth neonatal outcomes. Pregnancies obtained by frozen embryo transfers were excluded. This study was approved by the Institutional Review Board of the hospital. All procedures in the *Materials and Methods* section were compliant with ethical guidelines approved by the Ethical Committee.

### ICSI procedures and embryo culture

The procedures for ICSI have been described previously^15^. Briefly, during the ICSI processing, cumulus cells and the corona radiata of the oocytes were removed by brief exposure to hyaluronidase 2–3 hours after retrieval; ICSI was performed on metaphase II oocytes as observed under an inverted microscope. Then, the fertilized oocytes were continuously cultured in G1 medium (Vitrolife) for 2 more days. All of the embryos from PESA, TESA, and ICSI cycles were checked on the morning of day 3 after oocyte retrieval. Unless the quality of the embryo was very poor (>50% fragments or three or fewer cells on day 3, ET cancelled), fewer than two best quality embryos were usually transferred on day 3 after oocyte retrieval, according to the protocol developed by Chinese legislation^16^.

### Outcome measures

The method of outcome measures have been described previously^15^. Serum hCG was used to determine a biochemical pregnancy 14 days after ET; this level was subsequently tested serially to monitor the rise in titers. A clinical pregnancy was defined as the presence of a gestational sac with fetal heart activity on ultrasound examination 4–5 weeks after ET. The neonatal outcome data were obtained by telephone interview of the parents after delivery. The information on gestational weeks, sex, birth weight, and congenital birth defects was collected.

### Congenital malformations

Classification of the malformations was in accordance with European Surveillance of Congenital Anomalies (EUROCAT, http://www.eurocat-network.eu). The major malformation is classified as it has functional consequences or is in need of surgical correction. All others are classified as minor malformation. If the available data were insufficient for classification, the malformation was classified as major^11^.

### Statistical analysis

All data analyses were performed using the SPSS (version 13.0). The parameters were compared for the epididymal sperm, testicular sperm and ejaculated sperm groups using one-way analysis of variance and Duncan’s multiple-range tests. Non-ejaculated sperm group and ejaculated sperm group data were compared using the nonparametric Mann–Whitney U-test. The differences in outcomes between groups were analyzed using chi-square tests. The odds ratio (OR) and 95% confidence interval (CI) of each of the comparisons were calculated. Statistical significance was defined as a P value of <0.05.

## Results

### Demographics/clinical background data

**Figure 1:**
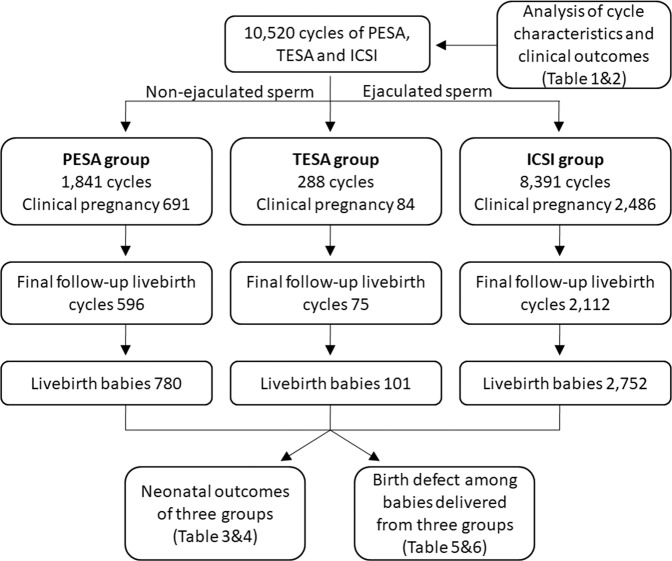
The flow chart of the study design.

A total of 10,520 cycles (PESA, n = 1,841; TESA, n = 288; ICSI, n = 8,391) were evaluated. The flow chart (Fig. 1) shows the design of this study. Baseline characteristics of ICSI cycle using extracted non-ejaculated (epididymal and testicular) sperm and ejaculated sperm are shown in Table 1. The age, duration of infertility, basal FSH, number of oocytes retrieved and number of mature oocytes of non-ejaculated sperm group were significantly different from those in ejaculated sperm group. The clinical pregnancy rate and implantation rate of non-ejaculated sperm group (52.4% & 37.2%) were significant higher (P < 0.001) than ejaculated sperm group (43.2% & 29.8%).

**Table 1:** Clinical characteristics of ICSI cycle according to sperm origin.

Parameter	Non-ejaculated sperm			Ejaculated sperm	Non-ejaculated versus ejaculated sperm group
	Total	*Epididymal sperm*	*Testicular sperm*		
No. of cycles	2129	1,841	288	8,391	
Age (y)	28.9 ± 4.9	28.6 ± 4.7 a	30.4 ± 5.7 b	31.3 ± 5.2 c	<0.001
Duration of infertility (y)	4.0 ± 3.4	4.0 ± 3.4 a	4.2 ± 3.5 a	4.7 ± 3.6 b	<0.001
Duration of stimulation (d)	9.78 ± 1.69	9.75 ± 1.67	9.95 ± 1.79	9.71 ± 1.89	NS
Basal FSH (IU/L)	6.74 ± 2.5	6.60 ± 2.2 a	7.65 ± 3.6 b	7.06 ± 3.2 c	<0.001
No. of oocytes retrieved	13.4 ± 8.0	13.5 ± 8.0 a	12.6 ± 8.1 a	11.4 ± 7.6 b	<0.001
No. of mature oocytes	11.7 ± 6.5	12.0 ± 6.5 a	9.5 ± 5.6 b	9.2 ± 5.7 b	<0.001
No. of cycles transferred	1479	1,311	168	5,748	
No. of embryos transferred	1.96 ± 0.27	1.96 ± 0.27	1.92 ± 0.29	1.95 ± 0.34	NS
Clinical pregnancy rate (% per ET)	775 (52.4)	691 (52.7) a	84 (50.0) a,b	2,486 (43.2) b	<0.001
Implantation rate (%)	1079 (37.2)	959 (37.2) a	120 (37.2) a	3333 (29.8) b	<0.001

The parameters of the epididymal sperm, testicular sperm and ejaculated sperm groups were compared and analyzed by using one-way ANOVA. Within the epididymal sperm, testicular sperm and ejaculated sperm groups with different superscripts letters within rows differ significantly (P < 0.05).

There were 775 clinical pregnancies from non-ejaculated sperm group (691 from epididymal sperm and 84 from testicular sperm) and 2,486 clinical pregnancies from ejaculated sperm group (Table 2). The miscarriage rate per transfer (5.00% vs. 5.03%; OR, 0.99; 95% CI, 0.77–1.29), ectopic pregnancy rate per clinical pregnancy (2.45% vs. 2.74%; OR, 0.89; 95% CI, 0.53–1.50), induced abortion rate per clinical pregnancy (1.03% vs. 0.84%; OR, 1.22; 95% CI, 0.54–2.78) and fetal deaths per clinical pregnancy (0.13% vs. 0.24%; OR, 0.53; 95% CI, 0.06–4.44) were comparable between non-ejaculated sperm group and ejaculated sperm group (P > 0.05). The live delivery rate per transfer of non-ejaculated sperm group was higher than that of ejaculated sperm group (45.4% vs 36.7%, P < 0.001). The percentage of singletons and twins between the two groups was similar and there was no triplets in all live deliveries. The male-female ratio of fetus was also similar between the two groups.

**Table 2:** Clinical outcomes of ICSI cycle according to sperm origin.

Parameter	Non-ejaculated sperm			Ejaculated sperm	Non-ejaculated versus ejaculated sperm group	
	Total	*Epididymal sperm*	*Testicular sperm*		P value	OR (95% CI)
No. of clinical pregnancies	775	691	84	2,486		
No. of miscarriages (% per ET)	74 (5.00)	67 (5.11)	7 (4.17)	289 (5.03)	NS	0.99 (0.77–1.29)
No. of ectopic pregnancies (% per clinical pregnancy)	19 (2.45)	18 (2.60)	1 (1.20)	68 (2.74)	NS	0.89 (0.53–1.50)
No. of induced abortions (% per clinical pregnancy)	8 (1.03)	8 (1.16)	0 (0)	21 (0.84)	NS	1.22 (0.54–2.78)
No. of fetal deaths (% per clinical pregnancy)	1 (0.13)	0 (0)	1 (1.20)	6 (0.24)	NS	0.53 (0.06–4.44)
No. of patients lost to follow-up (% per clinical pregnancy)	10 (1.29)	9 (1.30) a	1 (1.20) a	3 (0.12) b	<0.001	
No. of live deliveries (% per ET)	671 (45.4)	596 (45.5) a	75 (44.6) a	2112 (36.7) b	<0.001	
Singletons (% per live delivery)	461 (68.7)	412 (69.1)	49 (65.3)	1472 (69.7)	NS	
Twins (% per live delivery)	210 (31.3)	184 (30.9)	26 (34.7)	640 (30.3)	NS	
Triplets (% per live delivery)	0	0	0	0		
Male	431	382	49	1350		
Female	450	398	52	1402		
Sex ratio, male/female	0.96	0.96	0.94	0.96	NS	0.99 (0.85–1.16)

The parameters of the epididymal sperm, testicular sperm and ejaculated sperm groups were compared and analyzed by using one-way ANOVA. Within the epididymal sperm, testicular sperm and ejaculated sperm groups with different superscripts letters within rows differ significantly (P < 0.05).

### Neonatal characteristics

#### Singletons

Table 3 shows the neonatal outcomes of singleton gestation in ICSI cycle according to sperm origin. There were 1,933 babies born from the non-ejaculated and ejaculated sperm sourced embryos. No significant differences in delivery method, mean gestational age, preterm deliveries, mean and distribution of birth weight were observed between non-ejaculated sperm group and ejaculated sperm group (P > 0.05). In addition, all of the neonatal outcomes between epididymal sperm group and testicular sperm group were similar (P > 0.05). With similar extent, there were more girls than boys in both non-ejaculated sperm and ejaculated sperm group. The percentage of cesarean sections were extremely high (83.3%).

**Table 3:** Neonatal outcomes of singleton gestation in ICSI cycle according to sperm origin.

Parameter	Non-ejaculated sperm			Ejaculated sperm	Non-ejaculated versus ejaculated sperm group	
	Total	Epididymal sperm	Testicular sperm		P value	OR (95% CI)
No. of vaginal deliveries (%)	75 (16.7)	65 (15.8)	10 (20.4)	219 (14.9)	NS	
No. of cesarean sections (%)	386 (83.3)	347 (84.2)	39 (79.6)	1253 (85.1)	NS	
Mean gestational age, wk	38.1 ± 5.0	38.1 ± 4.9	37.6 ± 5.8	38.4 ± 3.5	NS	
No. of preterm deliveries (<37 wk) (%)	34 (7.38)	29 (7.04)	5 (10.2)	109 (7.40)	NS	1.00 (0.67–1.49)
No. of very preterm deliveries (<32 wk) (%)	3 (0.65)	2 (0.49)	1 (2.04)	9 (0.61)	NS	1.07 (0.29–3.95)
Live birth	461	412	49	1472		
Mean birth weight, g	3173 ± 636	3181 ± 614	3110 ± 804	3213 ± 586	NS	
Birth weight <1,500 g (%)	1 (0.22)	0 (0)	1 (2.04)	4 (0.27)	NS	0.80 (0.09–7.16)
Birth weight 1,500–2,499 g (%)	27 (5.86)	25 (6.07)	2 (4.08)	68 (4.62)	NS	1.28 (0.81–2.03)
Birth weight 2,500–3,999 g (%)	412 (89.4)	368 (89.3)	44 (89.8)	1303 (88.5)	NS	1.09 (0.78–1.53)
Birth weight ≥ 4,000 g (%)	21 (4.56)	19 (4.61)	2 (4.08)	87 (5.91)	NS	0.76 (0.47–1.24)
Male	219	194	25	719		
Female	242	218	24	753		
Sex ratio, male/female	0.90	0.89	1.04	0.95	NS	0.95 (0.77–1.17)

The parameters of the epididymal sperm, testicular sperm and ejaculated sperm groups were compared and analyzed by using one-way ANOVA. No significant differences were observed among three groups (P > 0.05).

### Twins

There were 420 twin babies (212 males and 208 females) born from the non-ejaculated sperm sourced embryos and 1,280 twin babies (631 males and 649 females) born from the ejaculated sperm sourced embryos (Table 4). Similar to singleton gestation, no statistically significant differences in neonatal outcomes were found between non-ejaculated and ejaculated sperm groups.

**Table 4:** Neonatal outcomes of twins gestation in ICSI cycle according to sperm origin.

Parameter	Non-ejaculated sperm			Ejaculated sperm	Non-ejaculated versus ejaculated sperm group	
	Total	Epididymal sperm	Testicular sperm		P value	OR (95% CI)
No. of vaginal deliveries (%)	9 (4.3)	9 (4.9)	0 (0)	23 (3.6)	NS	
No. of cesarean sections (%)	201 (95.7)	175 (95.1)	26 (100)	617 (96.4)	NS	
Mean gestational age, wk	36.5 ± 3.1	36.4 ± 3.3	36.8 ± 1.2	36.2 ± 3.5	NS	
No. of preterm deliveries (<37 wk) (%)	81 (38.6)	69 (37.5)	12 (46.2)	293 (45.8)	NS	0.74 (0.54–1.02)
No. of very preterm deliveries (<32 wk) (%)	4 (1.90)	4 (2.17)	0 (0)	24 (3.75)	NS	0.50 (0.17–1.45)
Live birth	420	368	52	1280		
Mean birth weight, g	2424 ± 550	2425 ± 565	2422 ± 430	2433 ± 539	NS	
Birth weight <1,500 g (%)	8 (1.90)	7 (1.90)	1 (1.92)	42 (3.28)	NS	0.57 (0.27–1.23)
Birth weight 1,500–2,499 g (%)	181 (43.1)	157 (42.7)	24 (46.2)	519 (40.5)	NS	1.11 (0.89–1.39)
Birth weight 2,500–3,999 g (%)	231 (55.0)	204 (55.4)	27 (51.9)	719 (56.2)	NS	0.95 (0.76–1.19)
Birth weight ≥ 4,000 g (%)	0 (0)	0 (0)	0 (0)	0 (0)		
Male	212	188	24	631		
Female	208	180	28	649		
Sex ratio, male/female	1.02	1.04	0.86	0.97	NS	1.05 (0.84–1.31)

The parameters of the epididymal sperm, testicular sperm and ejaculated sperm groups were compared and analyzed by using one-way ANOVA. No significant differences were observed among three groups (P > 0.05).

### Congenital anomalies

As shown in Table 5, similar rate of congenital malformations was found in non-ejaculated sperm group and ejaculated sperm group (1.48% vs. 1.49%; OR, 0.99; 95% CI, 0.53–1.86). Moreover, the comparison among the epididymal sperm, testicular sperm and ejaculated sperm groups showed that there was no significant difference in the incidence of congenital malformations of live births. The rate of boys with birth defect per total male live births from non-ejaculated sperm group were similar with that of ejaculated sperm group (1.48% vs. 1.19%; OR, 1.25; 95% CI,0.49–3.22). And, the male-female sex ratio in congenital malformations showed that non-ejaculated sperm group (0.86, 6/7, boys/girls) were higher than ejaculated sperm group (0.64, 16/25, boys/girls). However, these differences were not statistically significant (P > 0.05).

**Table 5:** Incidence of malformations of babies delivered from epididymal sperm, testicular sperm and ejaculated sperm groups.

Parameter	Non-ejaculated sperm			Ejaculated sperm	Non-ejaculated versus ejaculated sperm group	
	Total	Epididymal sperm	Testicular sperm		P value	OR (95% CI)
Numbers of birth defect (% per total live birth)^a^	13 (1.48)	12 (1.54)	1 (1.00)	41 (1.49)	NS	0.99 (0.53–1.86)
Male with birth defect (% per total male live birth)^a^	6 (1.48)	5 (1.31)	1 (2.04)	16 (1.19)	NS	1.25 (0.49–3.22)
Female with birth defect (% per total female live birth)^a^	7 (1.64)	7 (1.76)	0 (0)	25 (1.78)	NS	0.92 (0.40–2.14)
Singletons with birth defect (% per total singletons live birth)^a^	9 (1.95)	8 (1.94)	1 (2.04)	14 (0.95)	NS	2.07 (0.89–4.82)
Twins with birth defect (% per total multiples live birth)^a^	4 (0.95)	4 (1.09)	0 (0)	27 (2.11)	NS	0.45 (0.16–1.28)

^a^The parameters of the epididymal sperm, testicular sperm and ejaculated sperm groups were compared and analyzed by using one-way ANOVA. No significant differences were observed among three groups (P > 0.05).

No significant differences were found in the rate of congenital malformations between non-ejaculated sperm group and ejaculated sperm group, neither for singletons nor twins (Table 5). However, in all live birth with congenital malformations, the ratio of singletons with birth defect in non-ejaculated sperm group (69%, 9/13) was significantly higher (P < 0.05; OR, 4.34; 95% CI,1.13–16.62) than that in ejaculated sperm group (34%, 14/41) (Data not shown).

Table 6 shows the type of congenital birth defects. The incidence of major birth defects were not significantly different among the epididymal sperm, testicular sperm and ejaculated sperm groups (P > 0.05). The risk of congenital heart disease was comparable (P > 0.05; OR, 1.36; 95% CI, 0.48–3.82) between epididymal sperm group (0.64%, 5/780) and ejaculated sperm group (0.47%, 13/2752).

**Table 6:** Types of malformations among babies delivered from epididymal sperm, testicular sperm and ejaculated sperm groups.

	Epididymal sperm	Testicular sperm	Ejaculated sperm
Q00-Q07 Nervous	2: Cerebral aplasia 1, Congenital craniocerebral malformation 1		2: Congenital Hydrocephalus 1, Cerebral infarction 1
Q10-Q18 Eye, ear, face, neck			4: Ear pinna aplasia
Q20-Q28 Cardiovascular	5: Congenital heart disease		13: Congenital heart disease
Q30-Q34 Respiratory	1: Congenital lung aplasia	1: Congenital lung aplasia	5: Congenital lung aplasia
Q38-Q45 Gastrointestinal	2: Cleft lip/palate 1, Congenital intestinal atresia 1		6: Cleft lip 2, Cleft lip/palate 1, Congenital intestinal aplasia 1, Congenital choledochus dilatation 1, Congenital anus atresia 1
Q50-Q64 Genitourinary system	1: Hydronephrosis		4: Hydronephrosis 1, Congenital cyst of kidney 2, Testicular hypoplasia 1,
Q65-Q79 Musculoskeletal	1: Finger malformation		6: Finger malformation 5, Strephenopodia 1
Q80-Q89 Other			1: Hydrothorax
Minor birth defects total (% per total live birth)^a^	1 (0.13)	0 (0)	6 (0.22)
Major birth defects total (% per total live birth)^a^	11 (1.41)	1 (0.99)	35 (1.27)

^a^The parameters of the epididymal sperm, testicular sperm and ejaculated sperm groups were compared and analyzed by using one-way ANOVA. No significant differences were observed among three groups (P > 0.05).

## Discussion

This study included 881children born after ICSI using non-ejaculated (epididymal or testicular) sperm and 2,752 children using ejaculated sperm. Children born after an ICSI treatment with extracted non-ejaculated sperm showed no evidence of increased risks for miscarriages and malformations problems. They also did not differ significantly in birth weight, preterm deliveries, gestational age and sex ratio compared with those using ejaculated sperm. Consistent with other reports no significant difference regarding birth parameters and incidence of prematurity, low birth weight and very low birth weight rates was found in this study among the epididymal, testicular sperm and the ejaculated sperm groups^10,11,17–19^.

In the aspect of major birth defects, no significant difference was found between non-ejaculated sperm group and ejaculated sperm group. Similar with this result, published studies also showed no increased live birth risks according to the use of epididymal or testicular sperm^17,20^. Some meta-analyses concluded that higher rate of birth defects has been found for IVF and ICSI baby than baby conceived naturally but these risks did not differ between IVF and ICSI^21,22^. Therefore, the risk of birth defects did not seem to be connected with the origin of sperm^10,23^.

Fedder *et al*., reported a high incidence of hypospadias (1.6%) in 187 boys conceived by non-ejaculated sperm^24^. Other studies found that the hypospadias risk of boys conceived using non-ejaculated sperm and ejaculated sperm was similar^12^. In our series, no hypospadias was found in 431 boys conceived using non-ejaculated sperm.

In our center, for patients with an azoospermia diagnosed with NOA by urologists, we used TESA to obtian sperm. Although a higher incidence of chromosomal anomalies could be detected in the NOA group because of severe male factor infertility^25^, no difference was found regarding the neonatal outcome and malformations measures between children of fathers with TESA and PESA. In a meta-analysis^26^, an increased risk of miscarriages with testicular sperm compared with epidiymal sperm (OR 1.47; 95% CI 1.12 to 1.93) was reported. In our study, the miscarriages rate between PESA and TESA group did not differ significantly. Overall, no significant higher congenital malformation risk was observed in the NOA compared with the OA group in our study. Another recent study reported similar results^11^.

It is worth noting that non-ejaculated sperm group had better clinical pregnancy rate (52.4% vs. 43.2%) and implantation rate (37.2% vs. 29.8%) than ICSI control groups. In addition, PESA group had the highest clinical pregnancy rate (52.7%) among PESA, TESA and ICSI control groups. In agreement with this results, previous studies from China analysed 3,106 fresh cycles which had undergone ICSI treatment with different source of sperm. They reported that the rates of clinical pregnancy (53.2% vs. 47.1%) and embryo implantation (34.3% vs. 29.0%) were significantly higher in PESA group compared to ICSI control groups^27^. Another research reported similar results from 1,732 ICSI cases using PESA, TESA and ejaculated sperm. The clinical pregnancy rate of PESA group was 54.8%, significant higher than TESA (47.7%) and ejaculated sperm group (46.7%)^28^. The fact that, in the ejaculated sperm with a high rate of DNA damage, the sperm existing DNA damage was much lower in the testis. These DNA damage found in ejaculated sperm results from change occurring at the post-testicular level. Despite the spermatozoa may be selectively influenced by these DNA damage during earlier developmental stage^29,30^. A recent meta-analysis indicated that sperm DNA fragmentation rates were lower in testicular sperm than in ejaculated sperm and that clinical outcomes, including live birth rates, were higher for men with confirmed post-testicular sperm DNA fragmentation when using testicular-ICSI rather than ejaculated-ICSI^31^. In addition, the mean age and FSH level of non-ejaculated sperm group were significantly better than that of ejaculated sperm group, which was also the reason for higher clinical pregnancy rate.

For the issue of sex ratio, we haven’t found any differences in gender rate between PESA (0.96), TESA (0.94) and ICSI with ejaculated sperm groups (0.96). However, there were more girls in the PESA group ICSI with ejaculated sperm groups. In the published data of our center^15^, the sex ratio of 2,778 children conceived from conventional IVF was 1.13. In the present study, the PESA (P < 0.05; OR 0.85; 95% CI 0.72 to 0.99) and ICSI with ejaculated sperm groups (P < 0.01; OR 0.85; 95% CI 0.77 to 0.94) were significantly lower than that of conventional IVF cycles. In other study they also found more girls after an ICSI treatment with ejaculated or non-ejaculated sperm. Published literature indicated^24^ that the male sex ratio in epididymal and testicular sperm group were significantly lower than that of conventional IVF group (without ICSI). A similar sex ratio between the OA group and NOA group was also showed in a Belgian research^12^. These results were confirmed by our study, where the male sex ratio of ICSI treatment was lower than that of conventional IVF and did not differ in the different sperm origin.

In conclusion, the results of the present study show that there was no statistically increased risk in neonatal outcomes of newborns in the ICSI treatment with epididymal or testicular sperm. Our study may provide information for consultation for ICSI treatment in PESA or TESA patients. However, the sample size of congenital malformations was small in our series. Hence, the statistical power may be limited to detect small difference. Additional long-term follow-up of studies are needed to further validate the safety and efficacy of ICSI treatment with epididymal or testicular sperm. Furthermore, as a systematic review suggested^10^, standard methodology need to be established for follow-up studies after ART, with a physical examination at birth and psychomotor assessment in childhood.
